# Identification of axolotl BH3-only proteins and expression in axolotl organs and apoptotic limb regeneration tissue

**DOI:** 10.1242/bio.036293

**Published:** 2018-08-15

**Authors:** Vesna Bucan, Claas-Tido Peck, Inas Nasser, Christina Liebsch, Peter M. Vogt, Sarah Strauß

**Affiliations:** Department of Plastic, Aesthetic, Hand and Reconstructive Surgery, Hannover Medical School, Carl-Neuberg-Strasse 1, 30625 Hannover, Germany

**Keywords:** Axolotl, Ambystoma mexicanum, BH3 only, Limb regeneration, Apoptosis

## Abstract

Like other urodela amphibians, axolotls are able to regenerate lost appendages, even as adults, rendering them unique among higher vertebrates. In reaction to the severe trauma of a lost limb, apoptosis seems to be primarily implicated in the removal of injured cells and tissue homeostasis. Little, however, is known about apoptotic pathways and control mechanisms. Therefore, here we provide additional information regarding the mechanisms of tissue degradation. Expression patterns of Bcl-2 family members were analyzed using reverse transcriptase-PCR, western blotting and immunofluorescence. In our study, we identified ten putative axolotl orthologs of the Bcl-2 family. We demonstrated that BH3-only proteins are differentially expressed in some axolotl organs, while they are expressed broadly in tail composite tissue and limb regeneration blastema. The importance of Bcl-2 family members is also indicated by detecting the expression of proapoptotic protein Bak in spatial congruence to apoptosis in the early stages of limb regeneration, while Bcl-2 expression was slightly modified. In conclusion, we demonstrate that Bcl-2 family members are conserved in the axolotl and might be involved in the tissue degradation processes that occur during limb regeneration.

## Abstract (precis)

**Summary:** In our study we identified ten putative axolotl orthologs of the Bcl-2 family. We demonstrate that Bcl-2 family members are conserved in the axolotl and are involved in tissue degradation processes during limb regeneration.

## INTRODUCTION

Unlike humans, urodela amphibians are able to regenerate appendages and organs like their limbs, tail, spinal cord, lenses and parts of their heart and brain. Missing body parts are replaced in a process called epimorphic regeneration, when terminal differentiated cells re-enter the cell cycle and proliferate. After injury, the first steps towards limb regeneration are characterized by immediate hemostasis and rapid wound closure; typically achieved by migrating epithelial cells after 24 h (Tank et al., 1976). Injured tissue is removed by histolyses, degradation of the extracellular matrix by matrix metalloproteinases and hydrolases and phagocytosis of cellular debris (Yang and Bryant, 1994). In the following days, the wound epidermis (WE) thickens to the inductive apical epithelial cap (AEC) which is indispensable for regeneration (Tank et al., 1976; Whited et al., 2011). Beneath the cap, a proliferative mesenchymal cell cone constitutes the regeneration blastema, which subsequently grows to replace the missing structures.

Apoptosis is important in morphogenetic processes, mostly in the limitation of cell populations, which at certain time points are no longer needed. This is also true in wound healing and regeneration. Both processes start with an inflammatory reaction which has to be reasonably limited by subsequent death of immune cells to avoid unwanted damage. Other examples include apoptosis of myofibroblasts and capillary endothelial cells in the course of tissue modulation during wound healing (Desmoulière et al., 1995). Reduced apoptosis has been connected to hypertrophic scar formation (Greenhalgh, 1998).

Apoptosis in amphibian limb regeneration is less well characterized. Published in 2000, a study by Mescher et al. investigated apoptosis in the early stages of limb regeneration in normal and denervated forelimbs. Their focus was put on the fact that denervation of amputation stumps resulted in distal-to-proximal regression of the stump. Consistently, the apoptotic index, which was defined by percentage of terminal deoxynucleotidyl transferase (TdT) dUTP nick end labeling (TUNEL)-positive cells counted on sections of distal stump tissue, was increasing steadily over time between days 7 and 11 post-amputation (Mescher et al., 2000).

Vlaskalin et al. stained apoptosis in tissue from growing amputated forelimbs of adult newts. In the early wound healing stage, they mostly observed apoptotic cells in the periost at the wound and in degenerating muscle tissue surrounding the bone. A weaker signal was observed in the wound epithelium. Apoptosis decreased over time with a little delay in the areas of muscle breakdown. In the later stages, apoptotic cells remained at a constant low level; about 1-2% of total cells. Apoptosis in interdigital mesenchyme might be not involved in digit demarcation (Vlaskalin et al., 2004) as has been observed in limb morphogenesis of mammalians (Salas-Vidal et al., 2001). In conclusion, apoptosis seems to be implicated mostly in the early stages of normal limb regeneration, indicating that the main reasons for apoptosis are removal of injured cells and control of immune response. Interestingly, Guimond et al. observed that bone morphogenetic protein (BMP)-2 overexpression in regenerating limbs is the cause of formation of hypomorphic limbs, due to increased cellular condensation and apoptosis (Guimond et al., 2010). While BMP-2 overexpression seems not to have a great impact on cell proliferation, BMP inhibition by Noggin resulted in decreased proliferation rates and a similar phenotype to BMP-2 overexpression. This indicates that balanced cell proliferation and apoptosis are crucial elements of patterning in limb regeneration. This hypothesis is supported by the observation that in regenerating retinas higher rates of apoptosis were also detected in later stages (Kaneko et al., 1999), which might indicate a more profound role of apoptosis in tissue homeostasis.

BH3-only proteins have been seen as effector proteins in apoptotic pathways, transmitting the death signal by binding the multidomain members of B-cell lymphoma (Bcl)-2 family proteins (reviewed by Lomonosova and Chinnadurai, 2008). Nevertheless, expression of BH3-only proteins during axolotl limb regeneration has never been investigated. To our knowledge, so far no reports exist about axolotl homologs of Bcl-2 family members. Our study was intended to identify axolotl BH3-only proteins and to demonstrate possible implication during limb regeneration.

## RESULTS AND DISCUSSION

### Identification of axolotl BH3-only proteins

In order to identify BH3-only proteins in axolotls (*Ambystoma mexicanum*), we performed keyword searches in the salamander EST database (http://salamander.uky.edu/ESTdb/). Additionally, we performed BLAST searches with the orthologous sequences from *Xenopus laevis* (respective *tropicalis*) and zebrafish (*Danio rerio*). All sequences used in this study are identified in the salamander database; listed in Table 1. Putative axolotl orthologs for Bad (Bcl-2 antagonist of cell death), Bid (BH3-interacting domain death agonist), Bim (Bcl-2-interacting mediator of cell death), Bmf (Bcl-2-modifying factor), Bnip3 (Bcl-2/adenovirus E1B 19-kDa protein-interacting protein 3) and Beclin were found, while no orthologs for Bik (Bcl-2-interacting killer), Harakiri (Hrk), Noxa and Puma (p53-upregulated modulator of apoptosis) could be identified. Interestingly, no sequences for Bik, Noxa and Puma were found for *Xenopus* either, with the exception of *Xenopus tropicales*, for which a hypothetical protein was identified as putative ortholog in BLAST searches (Table 1). It remains to be elucidated if the respective orthologs of Bik, Noxa and Puma can be identified in genomic exon scans or are lost from the amphibian line. Harakiri was not found in zebrafish, *Xenopus* or axolotl (Table 1) and might have evolved later in vertebrate history. The work group around Ashkenazi likewise identified no zebrafish Harakiri ortholog in their studies, confirming the notion that the Harakiri gene is restricted to higher vertebrates (Eimon and Ashkenazi 2010; Kratz et al., 2006).

**Table 1.**
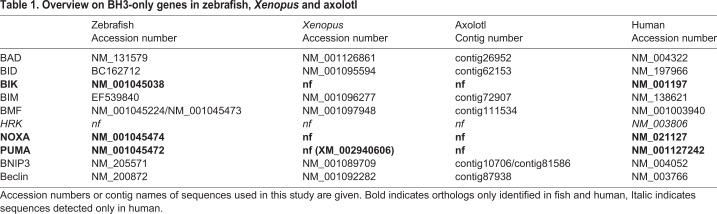
**Overview on BH3-only genes in zebrafish, *Xenopus*** **and axolotl**

Although cold-blooded animals like zebrafish and *Xenopus* are appropriate models in the study of apoptotic processes (Du Pasquier et al., 2006; Eimon and Ashkenazi 2010; Jette et al., 2008), little is known about the Bcl-2 family in non-mammalian vertebrates. Hsieh et al. cloned the zebrafish ortholog of Bad and demonstrated that it is involved in apoptosis. It was noted, however, that sequence identity to human, rat and mouse Bad was low, leading to differences at the N-terminus including the presence of only a single 14-3-3 binding site in contrast to two binding sites in mammalian sequences (Hsieh et al., 2003). Functional conservation was also shown for zebrafish Bim, Puma and Noxa in mouse embryonic fibroblasts, while zebrafish Bid was less active than expected, which may be due to functional divergence (Jette et al., 2008). Even less information is available for amphibian Bcl2 orthologs. Du Pasquier et al. analyzed Bid expression in larval and adult tissues. In functional analogy to the mammalian protein, Bid was localized to the mitochondrion, inducing a drop of mitochondrial membrane potential (ΔΨm) (Du et al., 2006).

### Analysis of BH3 domains

**Fig. 1.**
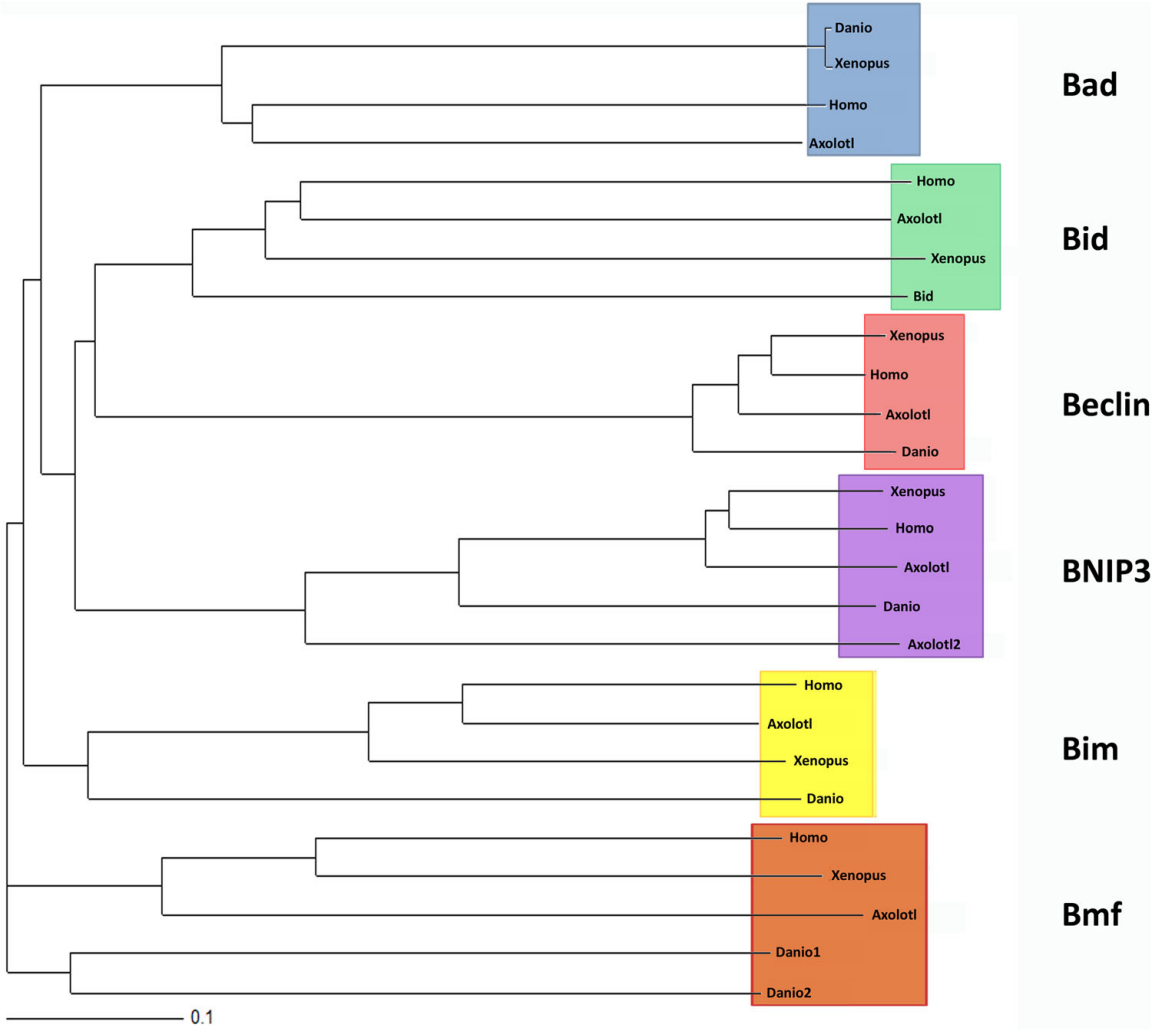
**Phylogenetic tree of BH3-only proteins.** Sequences found for zebrafish, *Xenopus*, human and axolotl were translated into amino acid sequences and aligned using ClustalW2 and T-coffee. A tree was generated and visualized. The scale bar indicates amino acid changes.

**Fig. 2.**
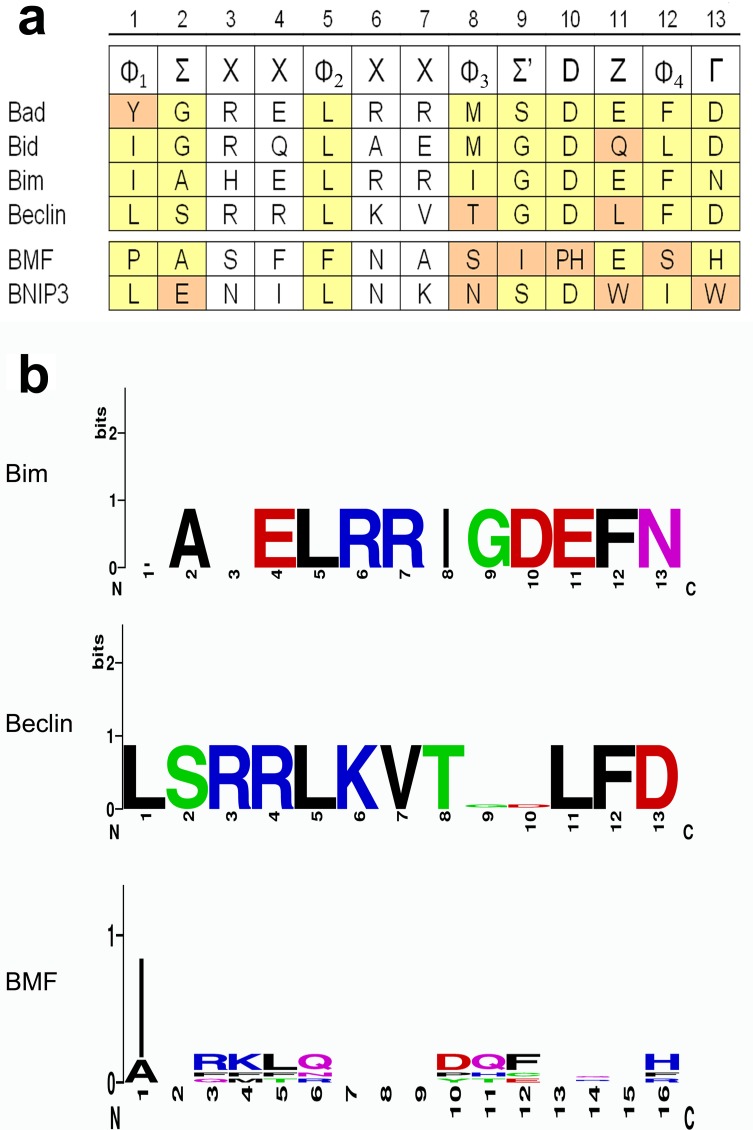
**Alignment of BH3 domains.** (A) BH3 consensus sequence as described by Day et al. (2008) is given in the first line. Amino acids identified as possible BH3 domains in putative axolotl orthologs are given below. Residues matching consensus are marked in yellow, while residues differing from the consensus are marked in orange. (B) WebLOGO plots based on the alignment of human, zebrafish, *Xenopus* and axolotl BH3 domains of Bim, Beclin and Bmf.

To confirm the identified sequence alignments, the deduced protein sequences were analyzed in a phylogenetic tree. All axolotl sequences group together with the corresponding sequences of zebrafish, *Xenopus* and human in the same clade, indicating that the true orthologs were included in the analysis (Fig. 1). Our study was not intended to look into the phylogenetic relation of the BH3-only proteins, taking into concern the limited sequence information used. In their more profound analysis, Aouacheria et al. stated that BH3-only proteins could not be defined as a clade due to their high sequence divergence outside the BH3 domain. They deduced that the multiple forms of BH3-only proteins may have evolved late to regulate cell death pathways in response to different stimuli (Aouacheria et al., 2005). BH3-only proteins have been described in *C. elegans* and several mammalian species but have not been identified in the annotation of *Drosophila* genome (Clavería and Torres, 2003). A 13-residue consensus motif has been proposed: Φ_1_ΣXXΦ_2_XXΦ_3_Σ’DZΦ_4_Γ with Φ representing a hydrophobic residue (e.g. Φ_2_ is usually a leucine); Σ is G, A or S; X is any amino acid; Z is often an acidic residue and Γ is a hydrophilic residue, e.g. N, H, D or Y (Day et al., 2008). With alignments to human and zebrafish sequences and analysis of consensus patterns, we were able to identify possible BH3 domains in all deduced amino acid sequences as indicated (Fig. 2A). The BH3 domain of axolotl Bim corresponds exactly to the proposed consensus sequence and orthologs of Bad, Bid and Beclin nearly match consensus, leading to the assumption that protein function might be conserved in axolotl. The sequence of Bmf and Bnip3 domain differ markedly from the consensus sequence, hence studies in appropriate model systems might confirm functional equivalence.

WebLOGOs were developed based on alignments of the BH3 domains identified in zebrafish, human, *Xenopus* and axolotl. As examples: BH3 domain of Bim is a typical BH3 domain, BH3 domain of Beclin is a less typical but highly conserved sequence and BH3 domain of Bmf is an untypical and less conserved sequence are shown in Fig. 2B.

### Expression in axolotl organs

**Fig. 3.**
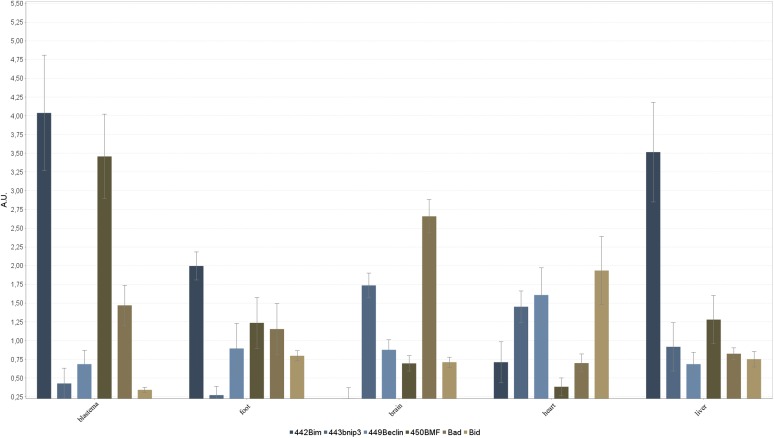
**RT-PCR analysis.** cDNAs prepared from the indicated tissues were used for amplification of BH3-only sequences using the primers as given in Table 2.

mRNA expression of BH3-only proteins and selected members of Bcl-2 family was investigated in blastema, foot, brain, heart and liver tissue in a semi-quantitative approach (Fig. 3). Transcripts were detected in different expression levels. All genomic DNA contamination controls were negative.

**Table 2.**
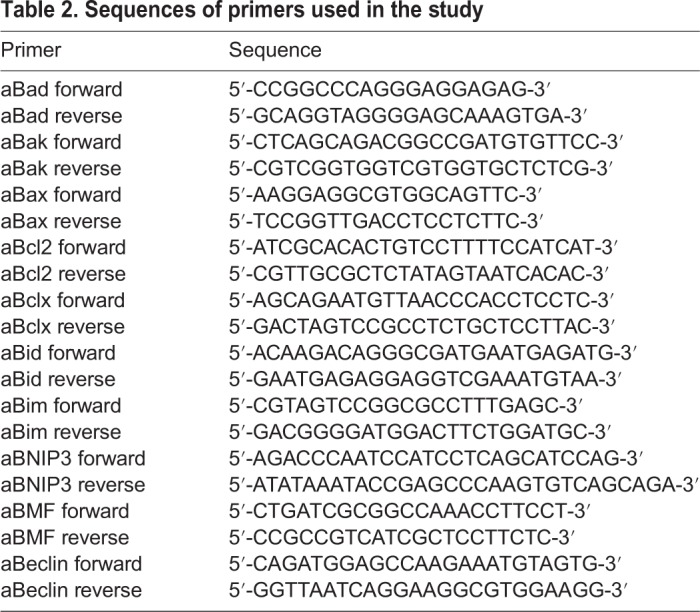
**Sequences of primers used in the study**

Bim expression levels were high in the blastema, foot and liver tissue, low in the heart and close to zero in the brain. The highest BMF expression level was found in the blastema tissue. Its expression was up to seven times higher compared to the other tested tissues. However, in the heart, expressions of Bnip3, Beclin and Bid were higher compared to the blastema, foot, brain and liver. The brain showed the highest expression of Bad. These expression patterns are consistent with the results of Hsieh et al., who also found a weak expression of Bad in zebrafish heart tissue and the results of Kratz et al., who detected low levels of Bid in the liver (Hsieh et al., 2003; Kratz et al., 2006). Kratz et al., however, detected Bid in zebrafish heart tissue and Du Pasquier et al. also found it in *Xenopus* heart tissue; this might be due to species variation between axolotl, *Xenopus* and zebrafish (Du Pasquier et al., 2006; Kratz et al., 2006).

**Fig. 4.**
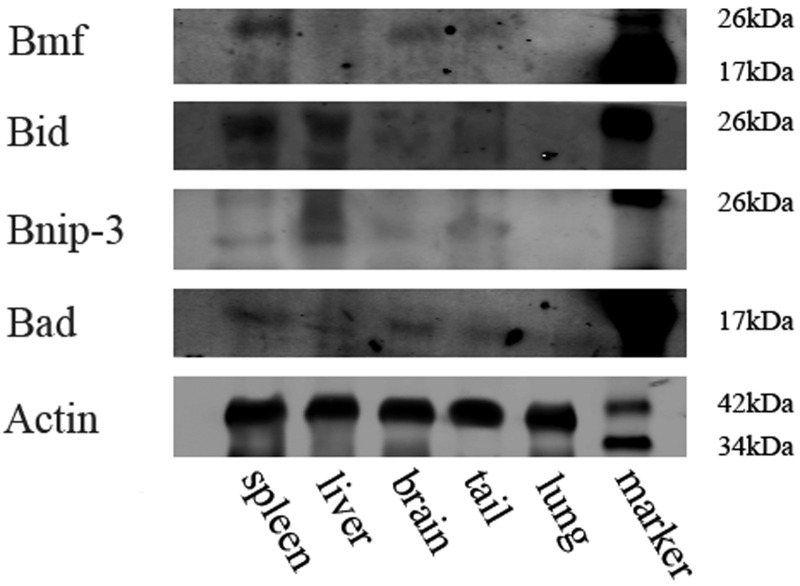
**Western blot analysis.** Whole axolotl organ lysates were subjected to western blotting followed by immunological detection of Bmf, Bid, Bnip-3, Bad and Actin.

Protein extracts were harvested from spleen, liver, brain, tail and lung tissue and analyzed for expression of Bmf, Bid, Bnip-3, Bad and Actin (Fig. 4). In spleen tissue bands corresponding to proapoptotic protein Bid could be detected. Bmf proteins were also shown in brain and very weakly in the liver, divergent from the PCR results. All samples showed presence of Bad proteins in the tissue. None of the antibodies tested were able to detect Bmf, Bid and Bnip-3 protein expression in the lung. It has to be noted that no antibodies specific for axolotl Bcl-2 family members were available, so antibodies directed against mammalian proteins were used, which may have led to lower affinity in axolotl proteins.

### Expression in limb regeneration tissue

**Fig. 5.**
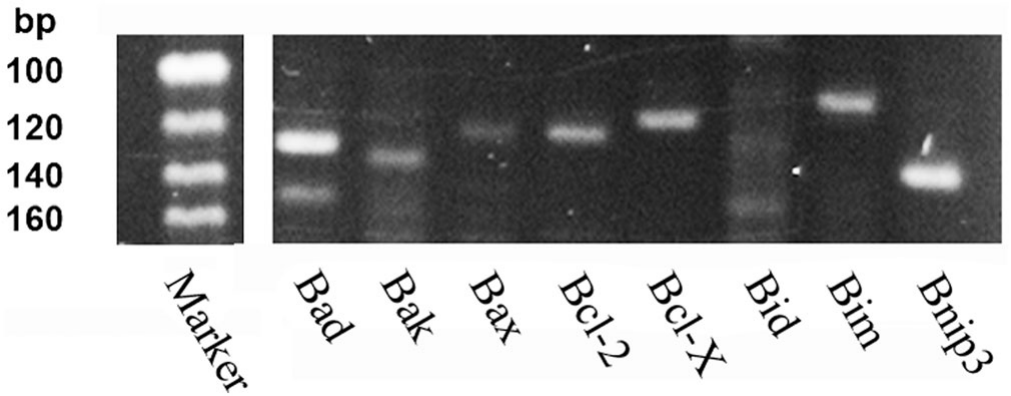
**Expression of Bcl-2 family members in axolotl limb regeneration blastema.** After reverse transcription of limb blastema derived RNA, gene-specific products were amplified based on the sequences retrieved from Sal-Site (http://www.ambystoma.org/) as indicated in Table 1.

In order to characterize a possible role for Bcl-2 family members in limb regeneration, we searched for expression in axolotl limb blastema. Transcripts of all identified multidomain and BH3-only family members were found in limb blastema. Genomic DNA controls were negative. Faint bands were detected for proapoptotic proteins Bak and Bax and for BH3-only protein Bid. The strongest bands were observed for antiapoptotic multidomain proteins Bcl-2 and Bcl-X and for BH3-only proteins Bim and Bnip3 (Fig. 5).

**Fig. 6.**
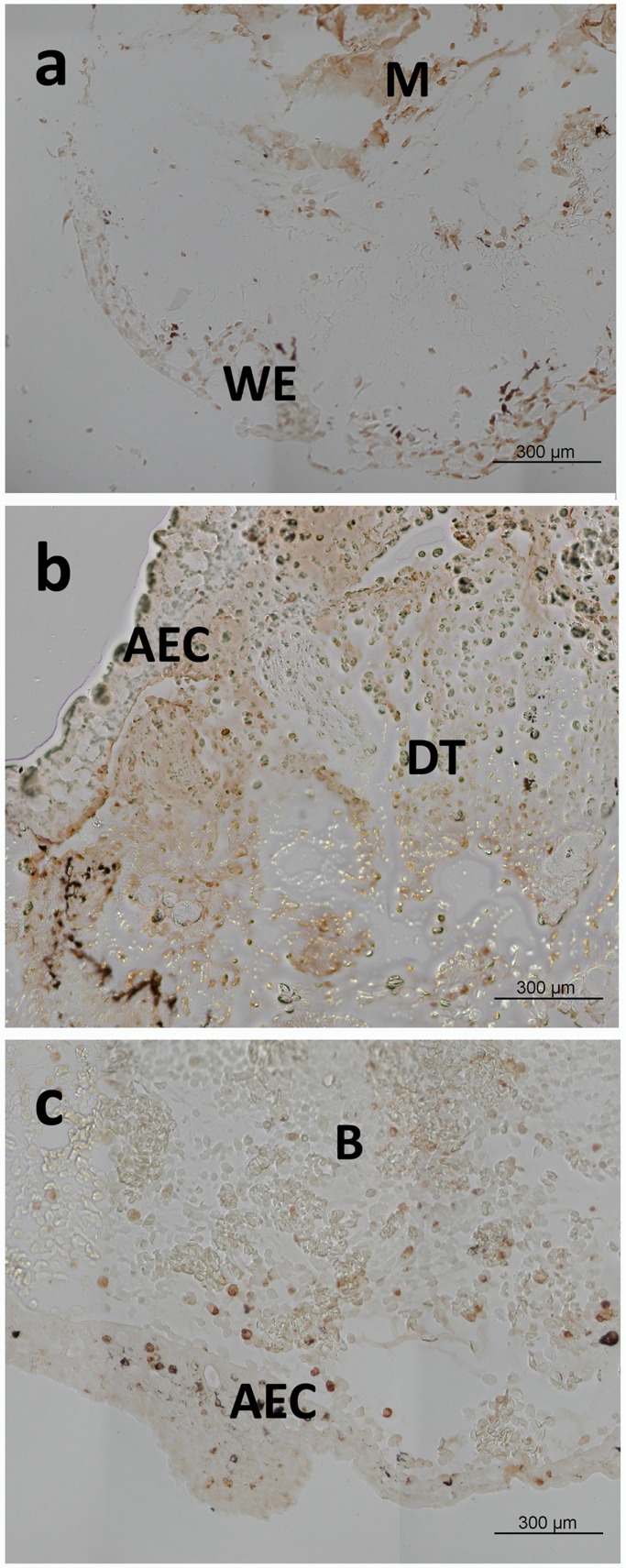
**TUNEL staining of early limb regeneration stages.** (A) Wound closure after 48 h. Apoptosis is stained in wound epithelium (WE), muscle (M) and periost near the amputation site. (B) Dedifferentiation stage (DT) after 5 days. Apoptosis is enhanced in the mesenchymal tissue beneath the thickened wound epidermis (apical epithelial cap, AEC). (C) In the mid-bud stage, only single apoptotic cells are observed in AEC and regeneration blastema (B).

Localization of Bak, Bcl-2 and Bid as examples for each type of Bcl-2 family subtype was investigated in tissue sections of three early states of axolotl limb regeneration. In the wound healing stage, single TUNEL positive cells are observed in the newly formed WE, in the muscle (M) and the periost tissue adjacent to the amputation site (Fig. 6A). In the next phase of limb regeneration, when the wound epidermis has thickened to the AEC, TUNEL staining is massively enhanced in the dedifferentiating cell mass (DT) below the AEC, while TUNEL positive cells are rarely in the AEC itself (Fig. 6B). Later on in mid-bud stage, a regenerative tissue cap of mesenchymal cells develops, which is mostly free of apoptotic cells. In the AEC, only single apoptotic cells could be detected (Fig. 6C).

**Fig. 7.**
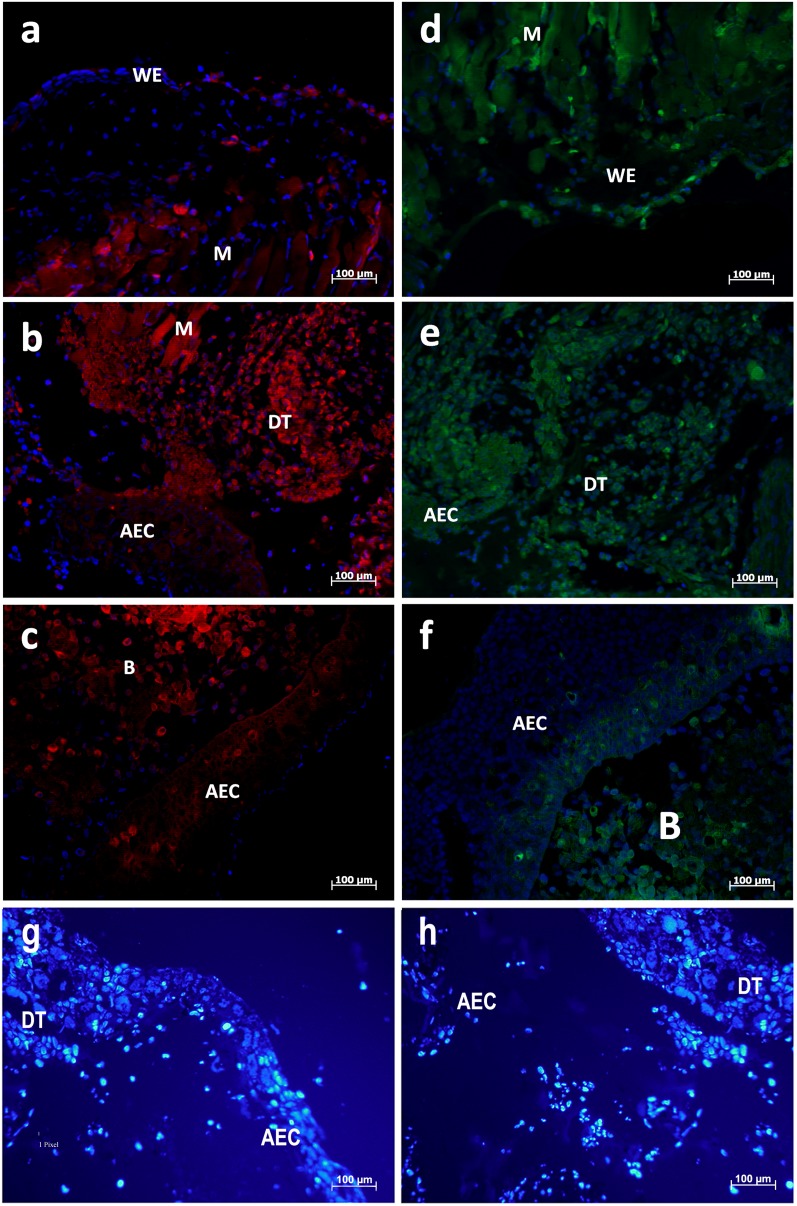
(A-C) Immunological detection of Bak (red fluorescence) in early limb regeneration stages. (D-F) Immunological detection of Bcl-2 (green fluorescence). Nuclei were counterstained with DAPI (blue fluorescence). (A) Single cells were found in the wound epithelium (WE) and adjacent muscle tissue (M) although a diffuse background staining is also found in the muscle tissue. (B) Bak is mainly expressed in the dedifferentiating tissue (DT), but single cells are also found in the apical epithelial cap (AEC) and muscle. (C) In the blastema (B) evenly distributed, single cells express Bak. In the AEC, most cells expressing Bak are found in the proximal cell layers. (D) Bcl-2 positive cells are regularly dispersed in WE and adjacent tissue. (E) Most Bcl-2 expressing cells are found in the DT layers next to the AEC. (F) Bcl-2 is expressed in the B and in the proximal layers of the AEC. (G,H) Tests with secondary antibodies.

As seen in Fig. 7, expression of proapoptotic protein Bak corresponds to a large extent to the regions where apoptosis occurs. Tests with only secondary antibodies were negative (Fig. 7G,H). Fig. 7A illustrates Bak expression at the wound healing stage. Single Bak expressing cells can be found in the wound epithelium while most Bak expression occurs in the muscle tissue near the amputation site. Analogous to the enhanced TUNEL staining at the phase of dedifferentiation, Bak expression is enhanced throughout muscle tissue, dedifferentiating tissue below the thickened AEC and in the AEC itself (Fig. 7B). In mid-bud stage, single Bak positive cells are found in the regeneration blastema and less so in the AEC (Fig. 7C). The spatial pattern of Bcl-2 expression resembles Bak expression (Fig. 7D-F). In their proteomic analysis of axolotl regeneration tissue, Rao et al. found that about 4% of proteins were apoptosis-related with upregulation ratios of 0.25 in the wound healing stage up to 1.4 in the late dedifferentiation/early blastema stage (Rao et al., 2009).

**Fig. 8.**
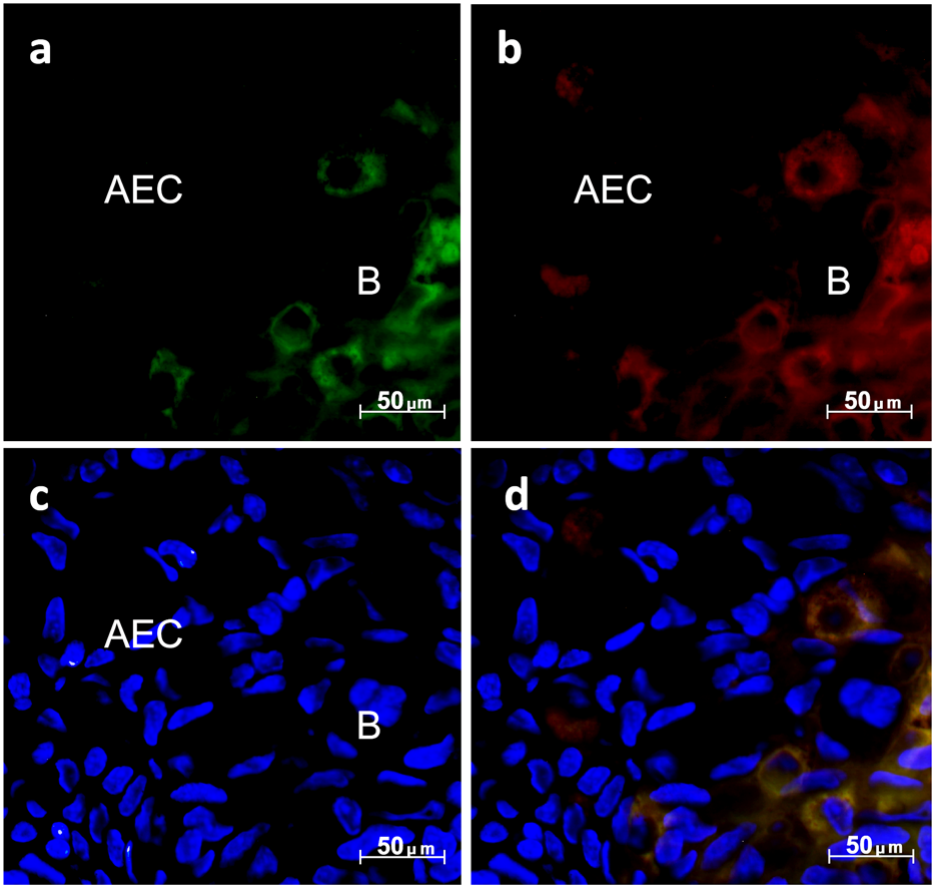
**Co-localization of Bcl-2 and Bid.** (A,B) In mid-bud stage, cells were stained for Bcl-2 expression (A; green fluorescence) and Bid expression (B; red fluorescence). (C) Nuclei were counterstained with DAPI (blue fluorescence). (D) Merged image. AEC, apical epithelial cap; B, blastema.

We have also shown that Bcl-2 and Bid are co-expressed in Bcl-2 positive cells in mid-limb bud blastema and AEC (Fig. 8). Preliminary tests with all conjugated secondary antibodies were carried out to eliminate unspecific bindings. While most cells in the AEC do not express Bcl-2 and Bid, the blastema cell layers beneath the AEC are more frequently found to express both (Fig. 8D). This is especially interesting as Bid belongs to the activator molecules of BH3-only proteins (Ren et al., 2010) which become activated upon cleavage within an unstructured loop (Li et al., 1998; Luo et al., 1998) and needs Bak for cytochrome c release (Wei et al., 2000). Functions beyond the regulation of apoptosis are also feasible (Yeretssian et al., 2011). In further studies, activation status, subcellular localization and complexion of Bid should be investigated to give insight into its participation in limb regeneration.

Interestingly, Lopez et al. published a study about thrombin-activated mitochondrial translocation of Bax, Bak and Bid in human platelets (Lopez et al., 2008). Proapoptotic Bcl-2 family members were activated within minutes after stimulation, and thrombin-dependent mitochondrial association was demonstrated. It might be interesting to investigate if part of the observed effect of thrombin activation on lens regeneration in newts and cell-cycle re-entry of cultured amphibian myotubes (Godwin et al., 2010; Imokawa and Brockes 2003; Loof et al., 2007; Tanaka and Brockes 1998) is also mediated by the Bcl-2 family, explicitly by BH3-only proteins.

## CONCLUSION

We were able to identify axolotl orthologs for proapoptotic multidomain proteins Bax and Bak, prosurvival Bcl-2 and Bcl-X and BH3-only proteins Bad, Beclin, Bid, Bim, Bmf and Bnip3. This is the most comprehensive assembly of Bcl-2 family members in the axolotl so far, although many family members still have to be identified. We investigated the expression pattern of BH3-only proteins in different axolotl tissues showing that broad expression can be found in foot, brain, heart and liver composite tissue and in dedifferentiated tissues like limb blastema. A functional conservation can be expected by expression pattern during the early stages of limb regeneration but has to be confirmed by further studies.

## MATERIALS AND METHODS

### Sequencing and bioinformatic analysis

Sequences were retrieved from the nucleotide database of the National Center for Biotechnology Information (NCBI; https://www.ncbi.nlm.nih.gov/) and Sal site (http://www.ambystoma.org). Accession Numbers can be found in Table 1. Multiple sequence alignment was performed using the ClustalW2 and T-coffee programs available at the EMBL-EBI server (http://www.ebi.ac.uk/Tools/msa/tcoffee/, http://www.ebi.ac.uk/Tools/msa/clustalw2/). A phylogenetic tree was visualized with Treeview (Page, 1996).

### Harvest of regeneration tissue

Animals were bred and kept at the Ambystoma Bioregeneration Center. The animals were housed under standard conditions and the experiments were carried out in accordance with the guidelines of the German Animal Welfare Act (TV-Nr.33.9-42502-04-10/0292). They were kept in tap water and fed on a specially developed diet (Axobalance; AquaTerratec, Bröckel, Germany). For amputations, young adult animals were anaesthetized in 0.01% ethyl-p-aminobenzoate (Sigma-Aldrich). Limbs were amputated proximal the wrist and regeneration tissue was staged according to Tank et al. (1976). Tissue samples were harvested several millimeters below the regeneration zone of three different animals in wound healing (day 2), dedifferentiation (day 5) and mid-bud stages (day 18). Samples were fixed in 3.7% formaldehyde, dehydrated and embedded in paraffin. For protein and RNA isolation, blastema tissue and axolotl organs were carefully prepared, snap frozen in liquid nitrogen and stored at −80°C until used.

### Tissue specific reverse transcription-polymerase chain reaction (RT-PCR)

RNA was isolated by using the Nucleospin RNA II Kit according to the user manual (Machery-Nagel, Düren, Germany). To confirm BH3-only transcripts in selected axolotl tissues, 2 µg total RNA was reverse transcribed in a reaction containing 0.25 mM dNTP, 1 µg random hexamer, 20 U recombinant StratascriptII with 1× Stratascript buffer supplied by the manufacturer (Stratagene, Amsterdam, The Netherlands). 2 µl of the reaction mixture was used in polymerase chain reaction (PCR) with specific primers (Table 2, synthesized by Eurofins MWG Operon, Ebersberg, Germany). Relative gene expression was determined by normalization of the ﬂuorescence intensity to 18S gene expression. Ampliﬁcation cycles were as following: 40 cycles at 94°C for 30 s, 65°C 30 s and 72°C 1 min. All experiments were carried out in triplicate and repeated at least at three independent times. The speciﬁcity of the Q-PCR products was proven by the appropriate melting curves (speciﬁc melting temperature). The data were analyzed using qRT-PCR data analysis software qbasePlus (Biogazelle, Zwijnaarde, Belgium). The amplification products were analyzed on 2% agarose gels supplemented with ethidium bromide.

### Western blot analysis

Axolotl tissue samples were transferred to RIPA buffer (10 mM Tris, pH 8, 150 mM sodium chloride, 1% Nonidet P-40, 0.5% sodium desoxycholat, 0.1% sodium dodecyl sulfate, 1 mM phenylmethylsulfonyl fluoride, 4 µg/ml aprotinin, 1 mM sodium orthovanadat) and lysed at 4°C for 48 h. Afterwards, the samples were sonicated and centrifuged at 16,600 ***g*** for 3 min to remove insoluble material. Protein content was determined using a Bradford assay (Pierce Coomassie Plus; Thermo Fisher Scientific) and used for normalization. SDS-polyacrylamide gel electrophoresis was followed by Western blotting to PVDF membrane (Immobilon FL; Millipore) using standard techniques. Membranes were blocked in Odyssey Blocking Buffer (LI-COR Biosciences, Lincoln, USA) for 1 h. Immunodetection was performed with primary antibodies diluted in Odyssey buffer supplemented with 0.1% Tween-20 as follows: rabbit polyclonal anti-Bid (Abcam): 1:1000, mouse monoclonal anti-Bcl-2: 1:300, mouse monoclonal anti-Bak (both Merck, Darmstadt, Germany): 1:1000. After incubation at 4°C overnight and extensive washing with TBST (Tris-based saline supplemented with 0.1% Tween) secondary antibodies (anti-mouse 800CW and anti-rabbit 680, respectively, LI-COR Biosciences) were added at a dilution of 1:20,000 and incubated at room temperature for 1.5 h. Near infrared signal were detected using an Odyssey scanner system equipped with appropriate software (LI-COR Biosciences).

### TUNEL staining

For detection of apoptotic DNA fragments, tissue sections were stained with Apoptag (Millipore) as recommended by the manufacturer. In brief, after deparaffinization, sections were treated with proteinase K (Sigma-Aldrich) and hydrogen peroxide, each time followed by washing steps. After equilibration sections were incubated with TdT enzyme diluted to working strength, as indicated in the manual, at 37°C for 1 h. After washing, samples were treated with peroxidase conjugated antibodies directed against digoxigenin at room temperature for 30 min. After extensive washing, reactions were detected with the addition of 3,3′-diaminobenzidine as peroxidase substrate. Reactions were stopped after 4 min when a brown color developed. Sections were mounted with Vectashield (Vector, Burlingame, USA) and analyzed on a digital microscope (Keyence, Neu-Isenburg, Germany). Sections treated with DNAse I (Machery-Nagel) before TUNEL staining were used as positive controls and sections left without TdT enzyme served as negative controls.

### Immunofluorescence

Tissue sections were deparaffinized and heated in antigen demasking solution (Vector). Followed by a wash step in phosphate buffered saline (PBS) for 5 min, the sections were treated with 0.1% Triton-X-100 in PBS for 5 min and blocked with 2% fetal calf serum in PBS. Antigens were detected by incubation with rabbit polyclonal antibodies directed against Bcl2, Bak or Bid at 4°C overnight, followed by extensive washing in PBS and incubated with conjugated secondary antibodies (Alexa Fluor 488 or 568 respectively; Molecular Probes, Leiden, The Netherlands) at 37°C for 30 min. Sections left without primary antibodies served as a negative control. After washing with PBS, samples were mounted with Vectashield, supplemented with 4′,6-diamidino-P-phenylindole (DAPI) and analyzed on a Zeiss Axiovert 200 M equipped with Apotome (Zeiss, Jena, Germany).
